# Intravenous lidocaine alleviates postherpetic neuralgia in rats via regulation of neuroinflammation of microglia and astrocytes

**DOI:** 10.1016/j.isci.2021.102108

**Published:** 2021-01-26

**Authors:** Lulin Ma, Juan Li, Junli Zhou, Dexin Zhang, Zhi Xiao, Tian Yu, Ying Li, Song Cao

**Affiliations:** 1 Department of Pain Medicine, Affiliated Hospital of Zunyi Medical University, Zunyi, China; 2 Department of Anesthesiology, Affiliated Hospital of Zunyi Medical University, Zunyi, China; 3 Guizhou Key Laboratory of Anesthesia and Organ Protection, Zunyi Medical University, Zunyi, China

**Keywords:** Cellular Physiology, Immunology, Neuroscience

## Abstract

This study aimed to explore the effects and possible mechanisms of intravenous lidocaine in postherpetic neuralgia (PHN) rats. Mechanical withdrawal thresholds and thermal withdrawal latencies were measured. Open field test, elevated plus maze test, and tail suspension test were used to assess anxiety- and depressive-like behaviors. Microglia and astrocytes in spinal dorsal horn (SDH), prefrontal cortex (PFC), anterior cingulate cortex (ACC), and hippocampus were analyzed. The expression of TNF-α, IL-1β, and IL-4 in SDH and serum were evaluated. Intravenous lidocaine alleviated mechanical allodynia and thermal hypoalgesia, downregulated the expression of TNF-α and IL-1β, and inhibited the activation of microglia and astrocytes in SDH. In addition, it reduced the activation of astrocyte but not microglia in PFC, ACC, and hippocampus. Intravenous lidocaine may relieve PHN by inhibiting the activation of microglia and astrocyte in SDH or by reducing the neuroinflammation and astrocyte activation in PFC, ACC, and hippocampus.

## Highlights

• Intravenous lidocaine alleviates PHN in rats
• Intravenous lidocaine inhibits microglia and astrocyte activation
• Intravenous lidocaine cannot relieve anxiety and depression in PHN rats

## Graphical Abstract

**Figure:**
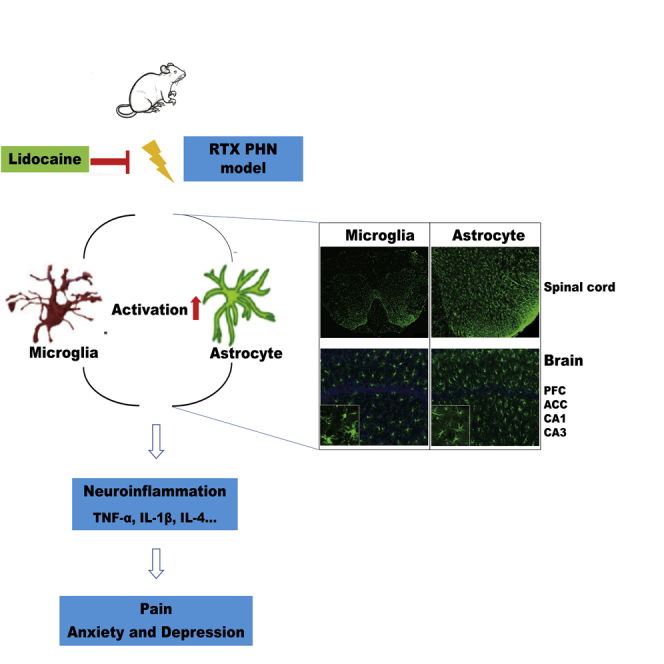

## Introduction

Neuropathic pain is a public health problem in modern society. About 30% of the world's population suffers from chronic pain, of which neuropathic pain accounts for about 6.9% (Bouhassira et al., 2008). Patients with chronic pain are more likely to have comorbidities such as sleep disorders, anxiety, and depression (McIlwrath et al., 2020), which further worsens patients' condition. Postherpetic neuralgia (PHN) is a typical neuropathic pain. It is an intractable pain in the lesion area lasting 3 months after varicella zoster virus infection (Scholz et al., 2019). Currently, for PHN treatment, effective therapeutic medicines or interventional procedures are still lacking, and studies of the pathogenesis and medications for PHN are urgently needed.

The pathogenesis of neuropathic pain is not clear enough now (Colloca et al., 2017); some studies have shown that neuropathic pain is closely related to the activation of glial cells in the spinal dorsal horn and brain (Tsuda et al., 2017; Zhou et al., 2019). Microglia and astrocytes are the pivotal cells that result in the development of acute and chronic pain after peripheral and central nerve injuries (Hains and Waxman, 2006; Ji et al., 2019). Multiple studies have shown the close relationship between neuropathic pain and the activation of glial cells in the spinal dorsal horn (Chen et al., 2019; Li et al., 2020), prefrontal cortex (PFC) (Fiore and Austin, 2019), anterior cingulate cortex (ACC) (Tsuda et al., 2017), and hippocampus (Barcelon et al., 2019; Liu et al., 2017). Furthermore, Ji et al. consider that neuropathic pain is related to central sensitization in the spinal cord, ACC, hippocampus, and other regions (Ji et al., 2018; Zhuo, 2007). The activation of microglia and astrocytes is mainly manifested by the increase of the cell number and the enlargement of cell body (Davis et al., 2017; Wang et al., 2017). In glial cells, changes for the expression of neuroinflammatory factors caused by transcriptional and post-transcriptional regulation are the key mechanisms that promote the occurrence of neuropathic pain. The activation and proliferation of microglia and astrocytes as well as the subsequent overproduction of inflammatory mediators, such as tumor necrosis factor alpha (TNF-α), interleukin-1β (IL-1β), etc., play an important role in the generation and maintenance of neuropathic pain (Grace et al., 2014; Ren and Dubner, 2016; Zhao et al., 2017).

Neuropathic pain is always accompanied by a variety of emotional disorders such as anxiety and depression. More than half of the patients with neuropathic pain have accompanying depression, cognitive impairment, or other emotional disorders (Liu et al., 2017; McIlwrath et al., 2020). Extensive studies have confirmed that the activation of microglia in the PFC, ACC, and hippocampus is closely related to anxiety and depression (Barcelon et al., 2019; Liu et al., 2019; Xu et al., 2017).

A combination of opioids, antiepileptics, and antidepressants is usually used to treat refractory neuropathic pain, although these drugs have complications such as respiratory depression, nausea, vomiting, and addiction. Lidocaine, the classic amide local anesthetic, has been widely used in clinical practice as a local anesthetic by local injection or by intravenous administration as an antiarrhythmic drug or anti-inflammatory drug. However, intravenous lidocaine has been proved to have a significant analgesic effect on various chronic pains, including neuropathic pains such as PHN and trigeminal neuralgia, and have no significant side effects (Yousefshahi et al., 2017). In addition, intravenous lidocaine reduced the opioid use and their side effects (Daykin, 2017; Reeves and Foster, 2017). Intravenous infusion of 2–5 mg/kg lidocaine has no obvious side effects, and only few patients have reported reactions such as drowsiness and dizziness (Iacob et al., 2018; Reeves and Foster, 2017), which were usually mild and short in duration (Iacob et al., 2018; Reeves and Foster, 2017). Intravenous lidocaine showed therapeutic effect on PHN, and the analgesic effect was still significant 6 h after infusion, with the analgesic time far exceeding its half-life (120 min) (Attal et al., 2004); Another study shows that intravenous lidocaine can enhance the efficacy of conventional treatment without significant side effect in patients with PHN (Tan et al., 2019).

In summary, intravenous lidocaine can relieve PHN, but its specific cellular and molecular mechanisms remain unclear. In this study we used the resiniferatoxin (RTX)-induced PHN rat model (Lei et al., 2016; Pan et al., 2003) and hypothesized that intravenous lidocaine alleviated PHN by inhibiting neuroinflammation caused by microglia and astrocytes in the spinal dorsal horn, PFC, ACC, and hippocampus.

## Results

### Intravenous lidocaine relieves mechanical allodynia and thermal hypoalgesia

**Figure 1:**
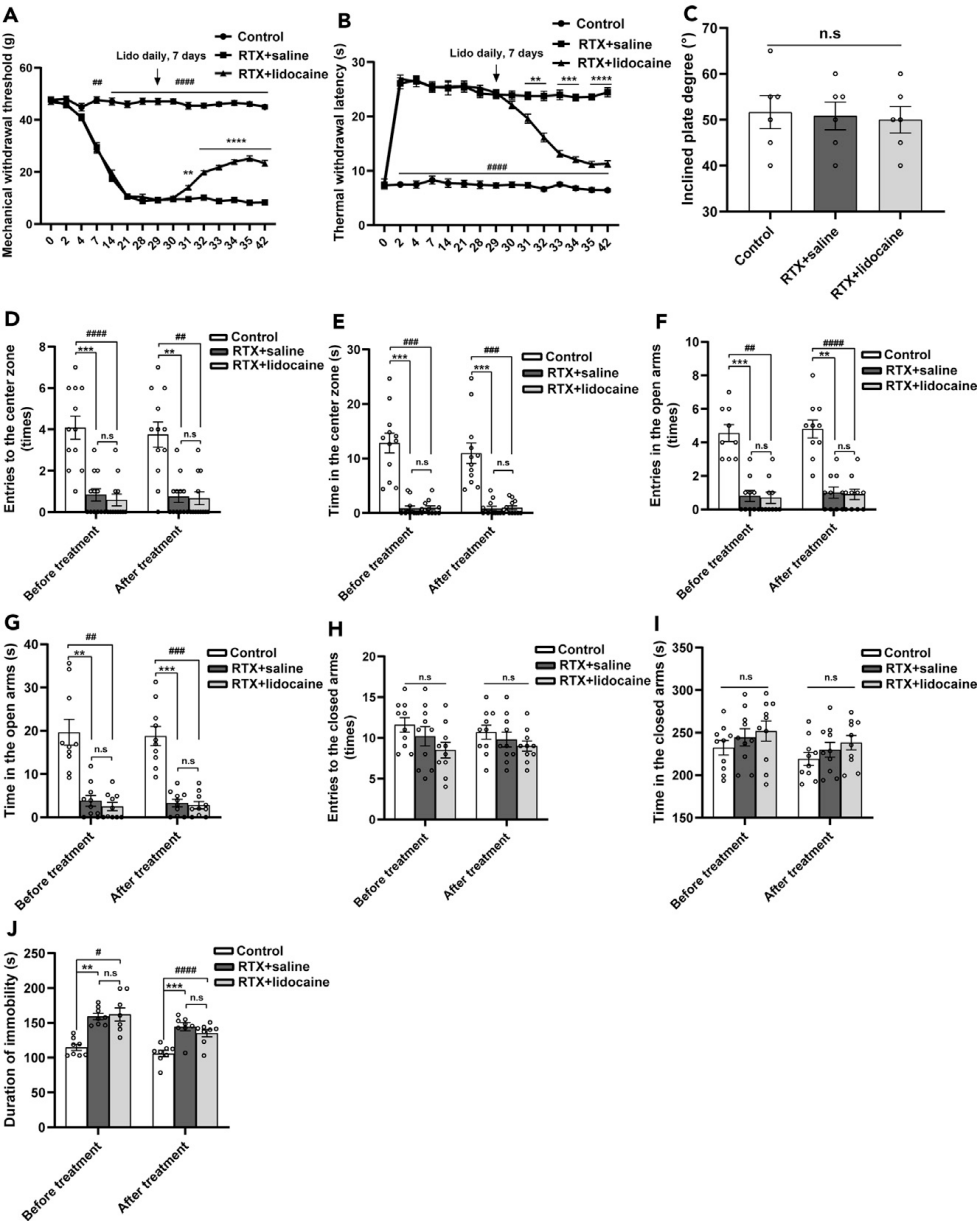
Intravenous lidocaine relieves mechanical allodynia and thermal hypoalgesia, but not anxiety- and depressive-like behaviors in PHN rats (A) Mechanical withdrawal thresholds tested with an electronic von Frey plantar aesthesiometer increased in RTX + lidocaine group compared with the RTX + saline group. ^##^p < 0.01, ^####^p < 0.0001, ∗∗p < 0.01, ∗∗∗∗p < 0.0001 versus RTX + saline group. (B) Thermal withdrawal latencies measured by a plantar thermal testing apparatus decreased in RTX + lidocaine group compared with RTX + saline group. ^####^p < 0.0001 versus RTX + saline group; ∗∗p < 0.01, ∗∗∗p < 0.001, ∗∗∗∗p < 0.0001 versus RTX + lidocaine group. (C) The inclined plate tests showed no difference in the incline degree among groups. (D) Entries to the center zone in the OFT. (E) Duration time in the center zone. Compared with the Control group, the entries and duration time in the center zone decreased in the RTX group. (F) Entries to the open arms in the EPM test. (G) Durations in the open arms. (H) Entries to the closed arms. (I) Durations in the closed arms. Compared with the Control group, in the RTX group, the entries and duration time in the open arms decreased, whereas no significant changes were detected in the closed arms. (J) The immobility time in the TST. Compared with the Control group, the immobility time of the RTX rats prolonged, whereas there was no significant difference between the RTX + saline group and RTX + lidocaine group. Data were expressed as mean ± SEM, n = 6 for mechanical withdrawal threshold tests, thermal withdrawal latency tests, and the inclined plate tests, n = 12 for OFT, n = 10 for EPM tests, and n = 8 for TST. ∗∗p < 0.01, ∗∗∗p < 0.001, ^#^p < 0.05, ^##^p < 0.01, ^###^p < 0.001, ^####^p < 0.0001; n.s = not significant. The above-mentioned statistical analyses were conducted using one-way ANOVA tests followed by Tukey's post-hoc tests.

The mechanical withdrawal thresholds of the RTX + saline rats significantly decreased 7 days after RTX injection and reached the lowest value on the 21^st^ day compared with the Control rats. On the 28^th^ day, the mechanical withdrawal thresholds still maintained at a low level. Compared with the RTX + saline group, the mechanical withdrawal thresholds of the RTX + lidocaine group started to increase after 3 days of lidocaine administration and continued to increase as time extended (Figure 1A).

Compared with the Control group, the thermal withdrawal latencies of the RTX + saline group significantly increased to the peak on the 2^nd^ day after RTX injection and maintained at the high level 28 days after RTX injection. Compared with the RTX + saline group, the thermal withdrawal latencies of the RTX + lidocaine group began to decrease after 3 days of lidocaine administration and continued to decrease as time extended (Figure 1B).

Twenty-eight days after RTX injection, inclined plate test was used to assess the motor function of the rats. Compared with the Control group, the inclined plate degree in the RTX + saline group and RTX + lidocaine group had no significant difference (Figure 1C).

### Intravenous lidocaine has no effects on anxiety- and depressive-like behaviors

PHN rats induced by RTX showed anxiety- and depressive-like behaviors 4 weeks after RTX injection. After 7 days of lidocaine administration, PHN rats still showed anxiety- and depressive-like behaviors. Intravenous lidocaine could not significantly alleviate anxiety- and depressive-like behaviors in PHN rats.

PHN rats show anxiety-like behaviors in the open field test (OFT). OFT was commonly used to evaluate anxiety-like behaviors in rats. Four weeks after RTX injection, compared with the Control group, in the RTX group, the entries and duration time in the central area decreased significantly. After 7 days of lidocaine administration, compared with the Control group, in the RTX group, the entries and duration time in the central area decreased significantly, but when compared with the RTX + saline group, in the RTX + lidocaine group, the entries and duration time in the central area showed no significant change (Figures 1D and 1E).

The elevated plus maze (EPM) tests were conducted to assess anxiety-like behaviors in rats. PHN rats show anxiety-like behaviors in EPM test. Four weeks after RTX injection, compared with the Control group, in the RTX group, the entries and duration time in the open arms decreased significantly, whereas no significant change of the entries and duration time was found in the closed arms. After 7 days of lidocaine administration, compared with the Control group, in the RTX group, the entries and duration time in the open arms decreased significantly, but not in the closed arms. Compared with the RTX + saline group, in the RTX + lidocaine group, the entries to the open and closed arms as well as the duration time in the open and closed arms showed no significant change (Figures 1F–1I).

The tail suspension test (TST) was used to evaluate depressive-like behaviors in the present study. PHN rats show depressive-like behaviors in TST. Compared with the Control group, the immobility time of the RTX rats prolonged significantly 4 weeks after RTX injection. After 7 days of lidocaine administration, compared with the Control group, the immobility time of the RTX rats still prolonged significantly; when compared with the RTX + saline group, it showed no significant change in the RTX + lidocaine group (Figure 1J).

### Intravenous lidocaine inhibits the expression of serum TNF-α and IL-1β, but has no significant effect on serum IL-4

**Figure 2:**
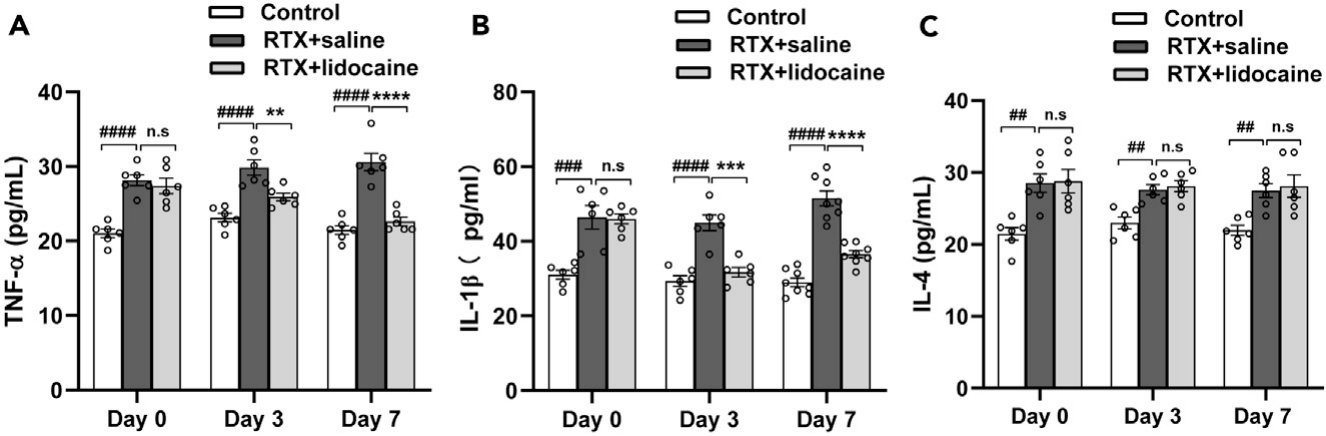
Intravenous lidocaine inhibits the expression of serum TNF-α and IL-1β, but has no significant effects on serum IL-4 (A) RTX increased serum TNF-α, whereas intravenous lidocaine inhibited the expression of serum TNF-α. (B) RTX increased serum IL-1β, whereas intravenous lidocaine inhibited the expression of serum IL-1β. (C) RTX increased serum IL-4, whereas intravenous lidocaine had no significant effects on serum IL-4. Data were expressed as mean ± SEM, n = 6–8. Statistical analyses consisted of one-way ANOVA tests followed by Tukey's post-hoc tests. ^##^p < 0.01, ^###^p < 0.001, ^####^p < 0.0001; ∗∗p < 0.01, ∗∗∗p < 0.001, ∗∗∗∗p < 0.0001; n.s = not significant.

Four weeks after RTX injection, before lidocaine administration, compared with the Control group, the expressions of serum TNF-α and IL-1β in the RTX + saline group were upregulated. Three days and 7 days after the first lidocaine administration, when compared with the RTX + saline group, in the RTX + lidocaine group, the expressions of serum TNF-α and IL-1β were downregulated. Compared with the Control group, the expression of serum IL-4 in the RTX + saline group was upregulated. However, there was no significant change of serum IL-4 expression before and after lidocaine administration between RTX + saline and RTX + lidocaine group (Figures 2A–2C).

### Intravenous lidocaine inhibits the activation of microglia and astrocyte and downregulates the TNF-α and IL-1β in spinal dorsal horn

**Figure 3:**
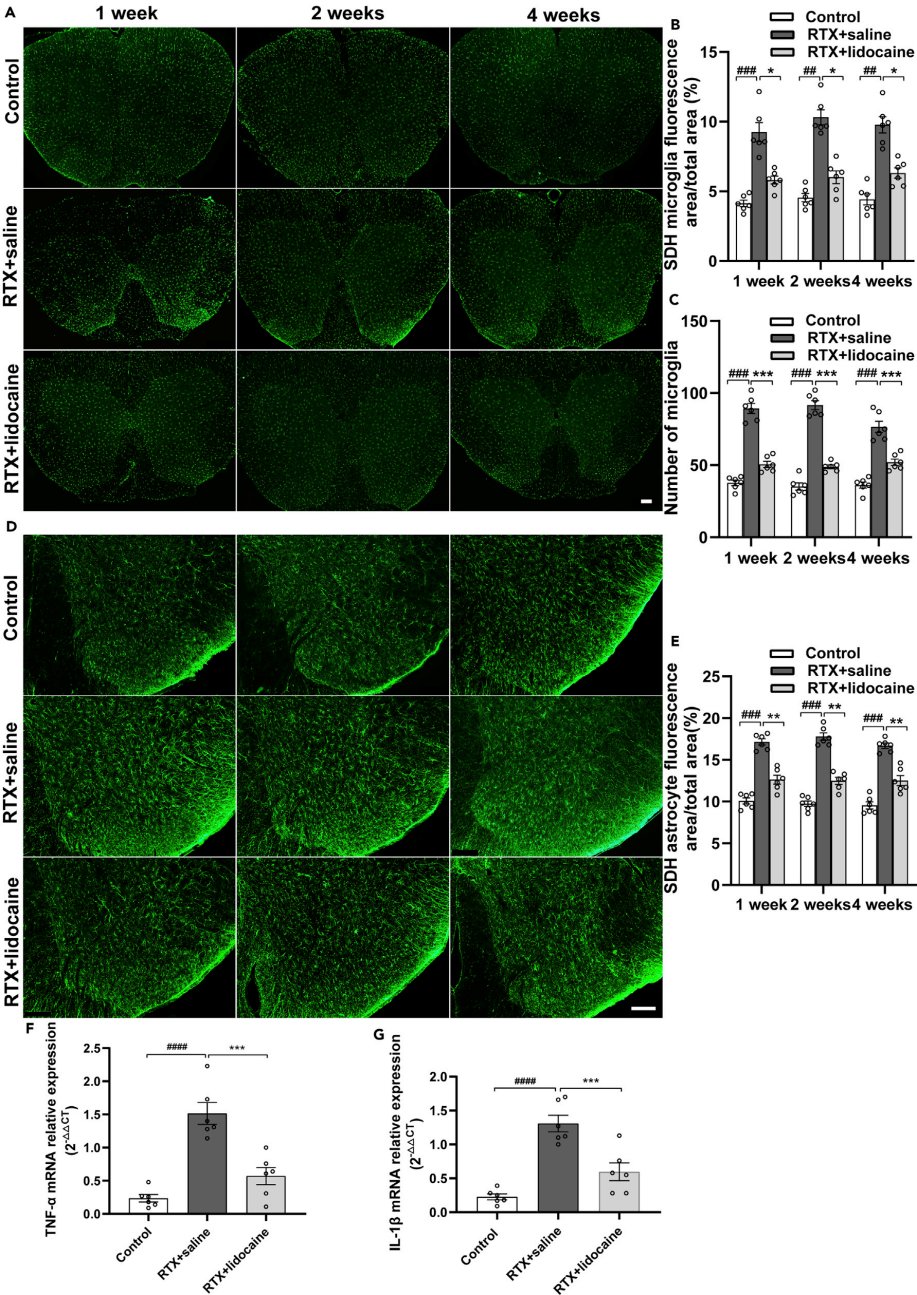
Intravenous lidocaine inhibits the activation of microglia and astrocyte, and the neuroinflammation in spinal dorsal horn (A) Immunofluorescence images of microglia in the spinal dorsal horn. (B and C) (B) Proportions of microglia-fluorescent area and (C) number of microglia in the spinal dorsal horn, which indicated that lidocaine infusion can reverse the RTX-induced activation of microglia. (D) Immunofluorescence images of astrocytes in the spinal dorsal horn. (E) Proportions of astrocyte-fluorescent area in the spinal dorsal horn, which indicated that lidocaine can reverse RTX-induced activation of astrocyte. (F) Intravenous lidocaine inhibited the expression of TNF-α mRNA in the spinal dorsal horn of RTX rats. (G) Intravenous lidocaine inhibited the expression of IL-1β mRNA in the spinal dorsal horn of RTX rats. The proportion and number of microglia and astrocytes were analyzed with ImageJ. Data were expressed as mean ± SEM. In (A–E), n = 6, 5 slices for each rat. In (F and G), n = 6. Statistical analyses consisted of one-way ANOVA tests followed by Tukey's post-hoc tests. Scale bars, 100 μm. ^##^p < 0.01, ^###^p < 0.001, ^####^p < 0.0001; ∗p < 0.05, ∗∗p < 0.01, ∗∗∗p < 0.001.

Immunofluorescence was used to evaluate the activation of microglia and astrocyte in the spinal dorsal horn. One week, 2 weeks, and 4 weeks after the first intravenous lidocaine administration (5 weeks, 6 weeks, and 8 weeks after RTX injection, respectively), compared with the Control group, more microglia were activated in the spinal dorsal horn of the RTX + saline group. Compared with the RTX + saline group, the activation of microglia in the RTX + lidocaine group was decreased (Figures 3A–3C).

One week, 2 weeks, and 4 weeks after the first intravenous lidocaine administration, compared with the Control group, more astrocytes were activated in the spinal dorsal horn of the RTX + saline group. Compared with the RTX + saline group, the activation of astrocyte in the RTX + lidocaine group decreased significantly (Figures 3D and 3E).

After the last lidocaine administration, the dorsal parts of the spinal cords were taken to detect the expression of TNF-α mRNA and IL-1β mRNA by PCR. Compared with the Control group, the expressions of TNF-α mRNA and IL-1β mRNA in the RTX + saline group were upregulated. Compared with the RTX + saline group, in the RTX + lidocaine group, their expressions were downregulated significantly (Figures 3F and 3G).

### Intravenous lidocaine inhibits the activation of astrocyte but not microglia in PFC

**Figure 4:**
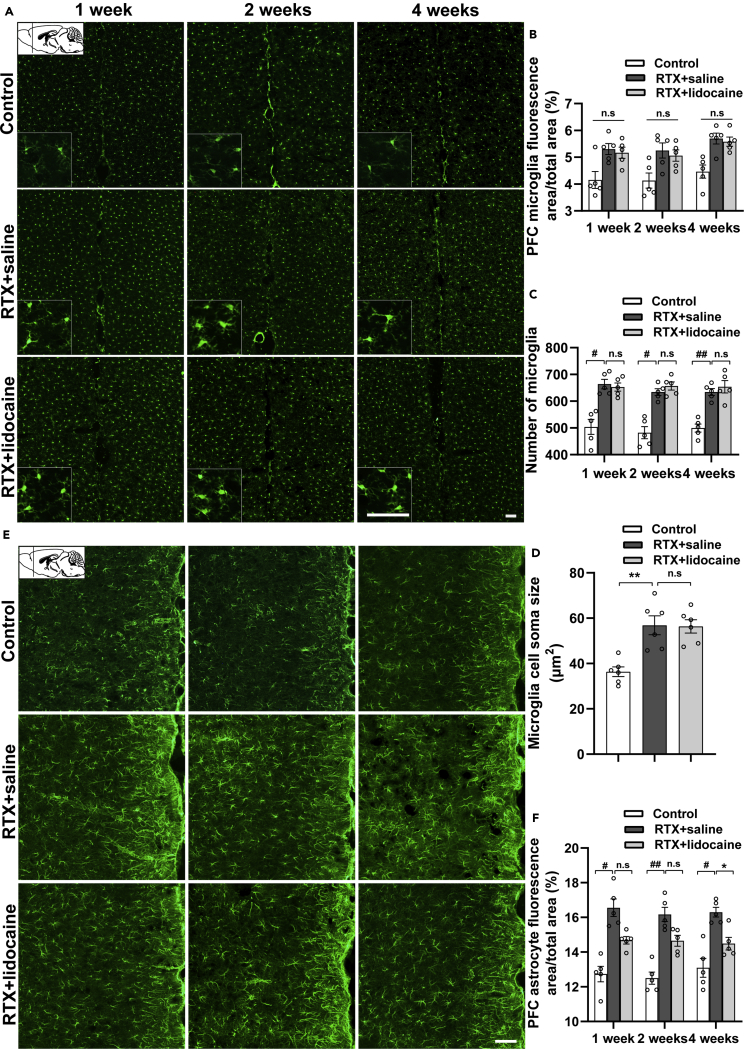
Intravenous lidocaine inhibits the activation of astrocyte, but not microglia in the PFC (A) Immunofluorescence images of microglia in PFC (bregma +2.68 mm). (B and C) (B) Proportion of microglia-fluorescent area and (C) number of microglia in PFC, which indicated that activated microglia by RTX cannot be inhibited by lidocaine. (D) The microglia soma sizes in PFC showed that activated microglia by RTX cannot be reversed by lidocaine. (E) Immunofluorescence images of astrocytes in PFC (bregma +2.68 mm). (F) Proportion of astrocyte-fluorescent area in PFC, which indicated that activated astrocytes by RTX can be reversed by lidocaine. The proportion, the number of microglia and astrocytes, and the cell soma sizes were calculated with ImageJ. Data were expressed as mean ± SEM, n = 6, 5 slices for each rat. Statistical analyses consisted of one-way ANOVA tests followed by Tukey's post-hoc tests. Scale bars, 50 μm. ^#^p < 0.05, ^##^p < 0.01, ∗p < 0.05, ∗∗p < 0.01, n.s = not significant.

One week, 2 weeks, and 4 weeks after the first intravenous lidocaine administration (5 weeks, 6 weeks, and 8 weeks after RTX injection, respectively), compared with the Control group, the RTX + saline group showed more microglia activation and enlarged cell soma size in PFC (bregma +2.68 mm). However, compared with the RTX + saline group, in the RTX + lidocaine group, no significant change was detected (Figures 4A–4D).

One week, 2 weeks, and 4 weeks after the first intravenous lidocaine administration, compared with the Control group, the astrocytes in the RTX + saline group was activated significantly in PFC (bregma +2.68 mm). Compared with the RTX + saline group, the RTX + lidocaine group showed no significant difference at 1 week and 2 weeks after the first lidocaine administration, whereas at 4 weeks after the first intravenous lidocaine administration, in the RTX + lidocaine group, the astrocyte activation decreased significantly (Figures 4E and 4F).

### Intravenous lidocaine inhibits the activation of astrocyte but not microglia in ACC

**Figure 5:**
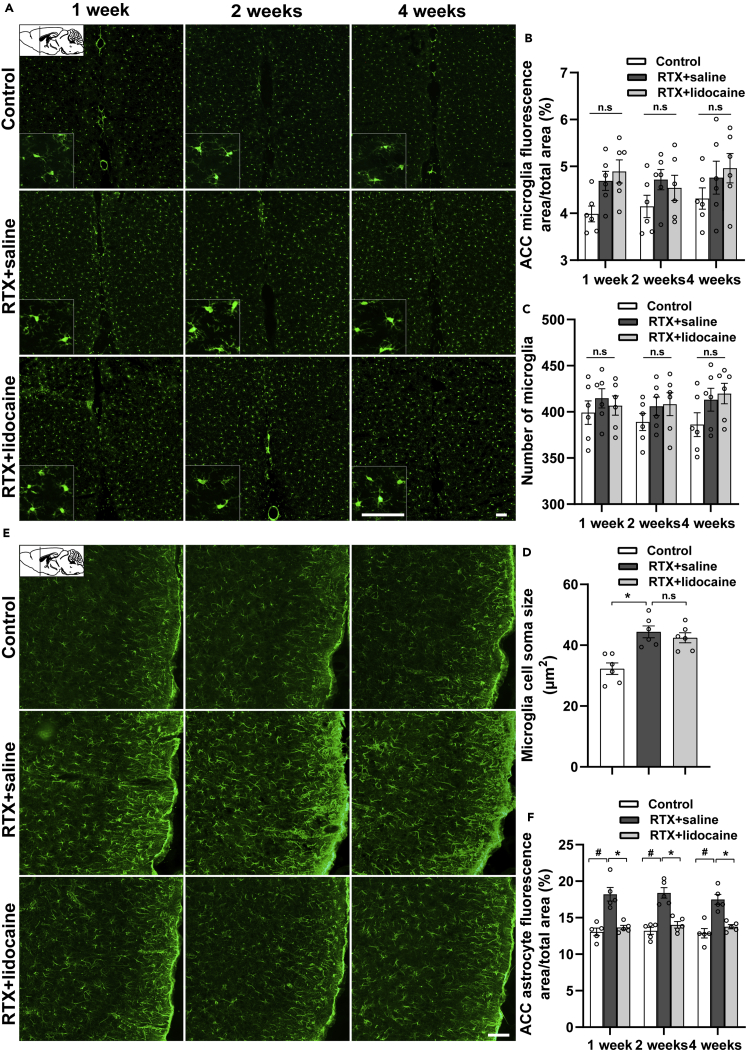
Intravenous lidocaine inhibits the activation of astrocyte but not microglia in ACC (A) Immunofluorescence images of microglia in ACC (bregma +1.18 mm). (B and C) (B) Proportion of microglia-fluorescent area and (C) number of microglia in ACC, which indicated that the activated microglia by RTX cannot be inhibited by lidocaine. (D) The microglia soma size comparisons in ACC, which indicated that the activated microglia by RTX cannot be reversed by lidocaine. (E) Immunofluorescence images of astrocytes in ACC (bregma +1.18 mm). (F) Proportion of astrocyte-fluorescent area in ACC, which indicated that the activated astrocytes can be reversed by lidocaine. The proportion, the number of microglia and astrocytes, and the cell soma sizes were calculated with ImageJ. Data were expressed as mean ± SEM, n = 5–6, 5 slices for each rat. Statistical analyses consisted of one-way ANOVA tests followed by Tukey's post-hoc tests. Scale bars, 50 μm. ^#^p < 0.05, ∗p < 0.05, n.s = not significant.

One week, 2 weeks, and 4 weeks after the first intravenous lidocaine administration (5 weeks, 6 weeks, and 8 weeks after RTX injection, respectively), compared with the Control group, the microglia in the RTX + saline group was activated, but the differences were not statistically significant in the ACC (bregma +1.18 mm). In addition, the cell soma sizes were enlarged. However, when compared with the RTX + saline group, in the RTX + lidocaine group, the activation of microglia showed no significant difference (Figures 5A–5D).

One week, 2 weeks, and 4 weeks after the first intravenous lidocaine administration, compared with the Control group, the astrocytes in the RTX + saline group were activated significantly, and when compared with the RTX + saline group, the activation of astrocyte in the RTX + lidocaine decreased in the ACC (bregma +1.18 mm) (Figures 5E and 5F).

### Intravenous lidocaine inhibits the activation of astrocyte but not microglia in hippocampus

**Figure 6:**
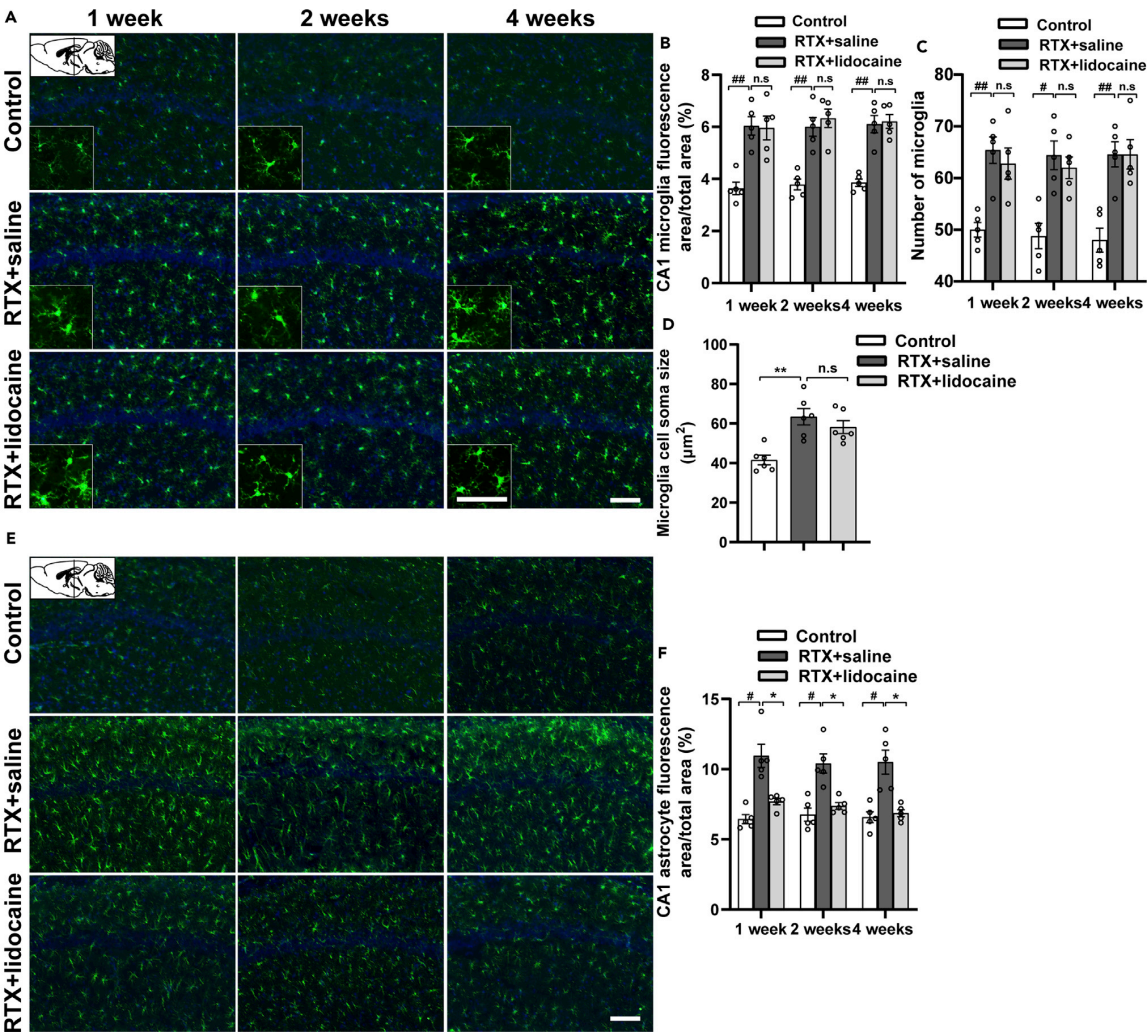
Intravenous lidocaine inhibits the activation of astrocyte but not microglia in CA1 (A) Immunofluorescence images of microglia in CA1 (bregma −1.58 mm). (B and C) (B) Proportion of microglia-fluorescent area and (C) number of microglia in CA1, which indicated that the activated microglia by RTX cannot be inhibited by lidocaine. (D) The microglia soma size comparisons in CA1, which indicated that the activated microglia cannot be reversed by lidocaine. (E) Immunofluorescence images of astrocytes in CA1 (bregma −1.58 mm). (F) Proportion of astrocyte-fluorescent area in CA1, which indicated that the activated astrocytes can be reversed by lidocaine. The proportion, number of microglia and astrocytes, and the cell soma sizes were calculated with ImageJ. Data were expressed as mean ± SEM, n = 5–6, 5 slices for each rat. Statistical analyses consisted of one-way ANOVA tests followed by Tukey's post-hoc tests. Scale bars, 50 μm. ^#^p < 0.05, ^##^p < 0.01, ∗p < 0.05, ∗∗p < 0.01, n.s = not significant.

**Figure 7:**
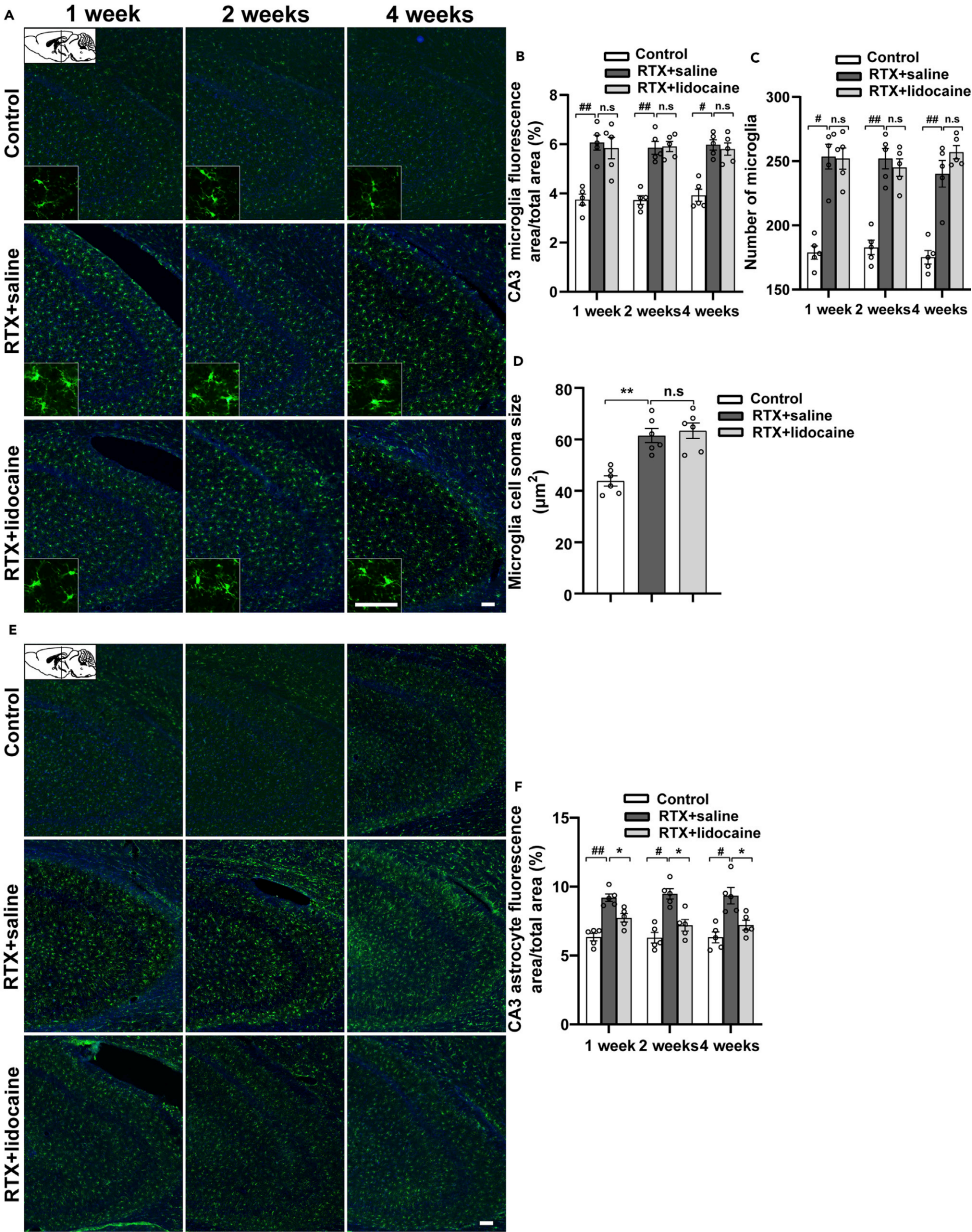
Intravenous lidocaine inhibits the activation of astrocyte but not microglia in CA3 (A) Immunofluorescence images of microglia in CA3 (bregma −1.58 mm). (B and C) (B) Proportion of microglia-fluorescent area and (C) number of microglia in CA3, which indicated that the activated microglia cannot be inhibited by lidocaine. (D) The microglia soma size comparisons in CA3, which indicated that the activated microglia cannot be reversed by lidocaine. (E) Immunofluorescence images of astrocytes in CA3 (bregma −1.58 mm). (F) Proportion of astrocyte-fluorescent area in CA3, which indicated that the activated astrocytes by RTX can be reversed by lidocaine. The proportion, number of microglia and astrocytes, and the cell soma sizes were calculated with ImageJ. Data were expressed as mean ± SEM, n = 5–6, 5 slices for each rat. Statistical analyses consisted of one-way ANOVA tests followed by Tukey's post-hoc tests. Scale bar, 50 μm. ^#^p < 0.05, ^##^p < 0.01, ∗p < 0.05, ∗∗p < 0.01, n.s = not significant.

One week, 2 weeks, and 4 weeks after the first intravenous lidocaine administration (5 weeks, 6 weeks, and 8 weeks after RTX injection, respectively), compared with the Control group, the microglia in hippocampus CA1 (bregma −1.58 mm, Figures 6A–6D) and CA3 (bregma −1.58 mm, Figures 7A–7D) of the RTX + saline group was activated and the soma sizes were enlarged. However, when compared with the RTX + saline group, the activated microglia of the RTX + lidocaine group had no significant difference.

One week, 2 weeks, and 4 weeks after the first intravenous lidocaine administration, compared with the Control group, in the RTX + saline group, the astrocytes in hippocampus CA1 (bregma −1.58 mm, Figures 6E and 6F) and CA3 (bregma −1.58 mm, Figures 7E and 7F) were activated significantly. When compared with the RTX + saline group, in the RTX + lidocaine group, the activation of astrocyte decreased.

## Discussion

In this study, intravenous lidocaine relieved the mechanical allodynia and thermal hypoalgesia, but had no significant effects on anxiety- and depressive-like behaviors in PHN rats. Intravenous lidocaine not only inhibited the activation of microglia and astrocytes in the spinal dorsal horn but also downregulated the expression of TNF-α and IL-1β in the serum and spinal dorsal horn. In addition, it inhibited the activation of the astrocytes, but not microglia in the PFC, ACC, and hippocampus. These data suggest a possible mechanism of PHN relieving by intravenous lidocaine infusion.

We found that continuous intravenous lidocaine for 7 days could relieve mechanical allodynia and thermal hypoalgesia in PHN rats. It had been reported that intravenous lidocaine (0.5 mg/kg/h or 2.5 mg/kg/h) relieved pain and allodynia in patients with PHN (Baranowski et al., 1999). Another study showed that intravenous infusion of 5 mg/kg lidocaine was effective in patients with PHN, and the analgesic effect was still significant 6 h after infusion, far exceeding its half-life (120 min) (Attal et al., 2004). Liu et al. showed that intravenous infusion of 5 mg/kg lidocaine reduced (or even stopped) the use of analgesics such as pregabalin and oxycodone in patients with PHN (Liu et al., 2018). Another study showed that on the basis of conventional PHN medication, intravenous infusion of 4 mg/kg lidocaine for 5 consecutive days enhanced the outcome of patients with PHN, which significantly decreased the pain intensity and the frequency of break-out pain (Tan et al., 2019). Consistent with the aforementioned studies, we find that in PHN rats, intravenous lidocaine relieves mechanical allodynia and thermal hypoalgesia.

In the present study, intravenous lidocaine inhibited the activation of microglia and astrocytes in the spinal dorsal horn. It also inhibited the neuroinflammation in the spinal dorsal horn. Spinal cord plays a critical role in the occurrence and development of neuropathic pain, and in recent years, more and more studies have confirmed that neuropathic pain results by the activation of glial cells in the spinal dorsal horn (Chen et al., 2019; Echeverry et al., 2017; Li et al., 2020). The activation of glial cells is mainly manifested by the increase in the number and the enlargement of the cell body (Davis et al., 2017; Wang et al., 2017). Moreover, the activated glial cells promote the release of pro-inflammatory cytokines (such as IL-1β, IL-6, and TNF-α) and induce neuroinflammation (Yang and Zhou, 2019). Neuroinflammation causes and aggravates neuropathic pain (Ji et al., 2018), which may be due to the production of pro-inflammatory factors leading to central sensitization of pain (Zhang et al., 2016). In the neuropathic pain model, the glial cells in spinal dorsal horn secreted a large number of cytokines, including pro-inflammatory factors such as TNF-α and IL-1β, which were essential for the development of neuropathic pain (Brifault et al., 2019; Clark et al., 2013; Mika et al., 2013). Disrupting the TNF signaling pathway and IL-1β signaling pathway could alleviate the pain hypersensitivity of rodent neuropathic pain (Clark et al., 2013). In summary, the activation of glial cells in the spinal dorsal horn and the subsequent neuroinflammation are an integral part of the occurrence and development of neuropathic pain. Lidocaine, as a classic local anesthetic, showed anti-inflammatory effects in several studies (Caracas et al., 2009; Hollmann and Durieux, 2000). For example, it inhibited lipopolysaccharide-induced inflammation in isolated microglia (Yuan et al., 2014). Our data indicate that intravenous lidocaine may alleviate the inflammation caused by the activation of glial cells in the spinal dorsal horn and then alleviate PHN in rats. Moreover, inflammation produced by glial cells plays an essential role in the generation of central sensitization and persistent pain, which could excite pain-related cells such as the wide-dynamic-range neurons (Ji et al., 2013). In addition, it has been reported that intravenous lidocaine could result in indirect effects of blocking nerve excessive and prolonged discharges, which also can relieve the pain (Dougherty et al., 1992).

We found that anxiety- and depressive-like behaviors appeared in PHN rats 4 weeks after RTX injection. However, intravenous lidocaine had no obvious effect on anxiety- and depressive-like behaviors. It did not affect the activation of microglia in the PFC, ACC, and hippocampus in PHN rats, either. Several studies have shown that neuropathic pain is often associated with comorbidities such as anxiety and depression (Barcelon et al., 2019; Dimitrov et al., 2014; Li et al., 2017). Among them, chronic neuropathic pain caused by peripheral nerve injury was accompanied with depressive-like behaviors and cognitive deficits (Dimitrov et al., 2014) and depressive-like behaviors appeared 4 weeks after chronic constriction injury (CCI) (Li et al., 2017). Moreover, the CCI model led to functional recovery and coping, or on the other hand to the emergence of debilitating and ongoing changes in affective state (Fiore and Austin, 2018). The mechanisms of depression caused by pain are not yet clear, but in patients with chronic pain and depression, functional magnetic resonance imaging (fMRI) has detected abnormal brain activity in the PFC, ACC and hippocampus (Fomberstein et al., 2013; Robinson et al., 2009). It may be closely related to the activation of microglia in brain areas such as the PFC, ACC, and hippocampus (Barcelon et al., 2019; Liu et al., 2019; Xu et al., 2017). Xu et al. found that in spared nerve injury (SNI) animals, neuropathic pain and depression coexisted, with microglia activation and amplified inflammatory cytokines in PFC. Minocycline administration reversed these abnormalities, indicating that microglia was closely related to neuropathic pain and depression (Xu et al., 2017). The activation of microglia in PFC and hippocampus and upregulated expression of TNF-α were induced in CCI mice, which showed depressive-like behaviors 8 weeks after CCI, suggesting that the activation of microglia in the brain was involved in the development of CCI-related affective disorders (Barcelon et al., 2019). In addition, the activation of hippocampal microglia may be a pivotal mechanism in the relationship between post-traumatic stress disorder (PTSD) and chronic pain, and inhibition of microglia activation may be a treatment target for chronic pain and PTSD (Sun et al., 2016). It is now generally accepted that neuroinflammation caused by microglia activation may be the main cause of anxiety and depression and other emotional disorders (Cao et al., 2019; Yirmiya et al., 2015). In the present PHN rat study and our previous fMRI study in patients with PHN (Cao et al., 2017), we believe that PHN can be accompanied by anxiety and depression, which may be related to the activation of microglia in the PFC, ACC, and hippocampus. Intravenous lidocaine did not significantly reduce the anxiety and depressive-like behaviors in PHN rats, which might be closely related to the failure to inhibit the activation of microglia in the PFC, ACC, and hippocampus. Similarly, it has been reported that intravenous infusion of 4 mg/kg lidocaine did not significantly improve the anxiety and depression status of patients with PHN (Tan et al., 2019). However, another study found that intravenous infusion of 5 mg/kg lidocaine not only reduced the amount of analgesics such as pregabalin and oxycodone but also improved the anxiety and depression in patients with PHN (Liu et al., 2018). The inconsistent outcomes may be related to the different evaluation time points after lidocaine administration.

Our data also showed that intravenous lidocaine could inhibit the activation of astrocyte but not microglia in the PFC, ACC, and hippocampus. As the headquarters of the nervous system, the brain contributes to the occurrence and development of neuropathic pain (Bantick et al., 2002). MRI detection suggested that multiple brain regions such as the hippocampus could be activated simultaneously during pain recognition (Bantick et al., 2002), whereas the activation of glial cells (microglia and astrocytes) in the PFC (Fiore and Austin, 2019), ACC (Tsuda et al., 2017), and hippocampus (Barcelon et al., 2019; Liu et al., 2017) could be contributors of neuropathic pain. Astrocytes could respond to central nervous system injury and disease, the so-called reactive astrocyte hyperplasia (Burda and Sofroniew, 2014). A large number of studies imply that brain glial cells may mediate the occurrence and development of neuropathic pain through inflammation (Hu et al., 2017; Liu et al., 2017; Sun et al., 2016). Therefore, it is possible that the activation of microglia and astrocytes in the PFC, ACC, and hippocampus contributes to PHN. In addition to affecting spinal dorsal horn glial cells, we find that intravenous lidocaine inhibits the activation of astrocyte in PFC, ACC, and hippocampus, but cannot inhibit the activation of microglia in these brain areas, suggesting that lidocaine cannot inhibit the microglial neuroinflammation in these brain regions. The other possible reason is that the medication time of intravenous lidocaine is too short (1 week) so that the changes of microglia have not been detected. Moreover, in addition to neuroinflammation, multiple receptors, channels, and transporters are expressed in glial cells and are regulated in different pain conditions (Ji et al., 2013). For example, in neuropathic pain condition, both p-p38 MAPK and p-ERK are increased in activated microglia and p-Jun-N-terminal kinase (p-JNK) is increased in GFAP-positive astrocytes (Lee et al., 2018). Chemokine receptors such as CX3CR1 are exclusively expressed in microglia, and CCL2 is mainly expressed in astrocytes (Ji et al., 2013). These indicate that microglia and astrocytes have different mechanisms of action in neuropathic pain. This may be another reason why intravenous lidocaine inhibits astrocyte activation but not microglia. In sciatic nerve ligation (SNL) mouse model, the emotional and cognitive changes during neuropathic pain were relatively separated from pain. When pain was properly treated, the emotional comorbidities may not be relieved accordingly (Dimitrov et al., 2014). SNL rats presented mechanical allodynia and depressive-like behaviors 7 days after modeling. Although kynurenine 3-monoxygenase inhibitor Ro 61-8048 significantly alleviated depressive-like behaviors in SNI rats, but had no significant effect on mechanical allodynia. The authors believed that although neuropathic pain was always accompanied by comorbidities such as anxiety and depression, they are caused by relatively different mechanisms (Laumet et al., 2017). Therefore, microglia may take part in the formation of emotional disorders such as anxiety and depression with different mechanisms from pain induction, which may also be the reason why intravenous lidocaine cannot inhibit the activation of microglia in the brain.

Interestingly, in the present study, intravenous lidocaine could inhibit the activation of microglia in the spinal dorsal horn, but not in the PFC, ACC, and hippocampus. Microglia activation in the brain and spinal cord is correlated with neuroinflammation (Cao et al., 2021). After peripheral nerve injury, TNF-α could differentially regulate synaptic plasticity in the hippocampus and spinal cord through microglia-dependent mechanisms (Liu et al., 2017). They thought that in the case of neuropathic pain, the activated glial cells may release different glial transmitters in the hippocampus and spinal dorsal horn (Liu et al., 2017). Thus, we speculate that the activation mechanisms of microglia between spinal dorsal horn and brain are different in neuropathic pain rat. Additionally, the mode of release of neurotransmitters by microglia is different in neuropathic pain environment. For example, in addition to mediating neuroinflammation, activated microglia also mediate other neurotransmitters, such as brain-derived neurotrophic factor (BDNF), a growth factor that could induce mechanical allodynia. After peripheral nerve injury, the level of BDNF decreased in the hippocampus, but increased in the spinal dorsal horn (Liu et al., 2017). This indicates that microglia in the spinal cord and in the brain respond differently in the neuropathic pain environment, which may be the reason why lidocaine acts differently on microglia activation in the spinal dorsal horn and in the brain.

### Limitations of the study

In this study, only two pro-inflammatory cytokines, i.e., TNF-α and IL-1β, were detected in the dorsal part of the spinal cord, whereas the anti-inflammatory cytokines had not been tested accordingly. In addition, in the PFC, ACC, and hippocampus, lidocaine's effects were only evaluated at the cellular level, but not at the molecular level. Furthermore, the differential responses of microglia to intravenous lidocaine in the spinal cord and brain need further investigation.

### Resource availability

#### Lead contact

Further information and requests for resources and reagents should be directed to and will be fulfilled by the Lead Contact, Song Cao (caosong4321@163.com).

#### Materials availability

This study did not generate new unique reagents.

#### Data and code availability

The data and codes reported in this study are available from the Lead Contact on request.

## Methods

All methods can be found in the accompanying Transparent Methods supplemental file.

## Supplemental Information

Document S1. Transparent methods and Table S1
