# Supplementary material for: Intravenous lidocaine alleviates postherpetic neuralgia in rats via regulation of neuroinflammation of microglia and astrocytes
Source: iScience. 2021 Jan 26;24(2):102108. doi: 10.1016/j.isci.2021.102108 (PMC7876569; doi:10.1016/j.isci.2021.102108)
Supplement: Document S1. Transparent methods and Table S1 [file mmc1.pdf]

iScience, Volume *24*

Supplemental Information

Intravenous lidocaine alleviates postherpetic neuralgia in rats via regulation of neuroinflammation of microglia and astrocytes

Lulin Ma, Juan Li, Junli Zhou, Dexin Zhang, Zhi Xiao, Tian Yu, Ying Li, and Song Cao

Transparent Methods

Animals

This study was approved by the Animal Care and Use Committees of Zunyi Medical University. Animal studies were performed in accordance with the Guide for the Care and Use of Laboratory Animals in China. Studies have shown that sex has an effect on pain and depressive-like behaviours(Liu et al., 2019; Sorge and Totsch, 2017), therefore, in the present study, only males were selected to control variables. Male Sprague-Dawley rats (6-8 weeks old, 200–250 g) were purchased from the Changsha Tianqin Biotechnology (Changsha, China). Three to four rats were accommodated in a cage at a constant room temperature of 23 ± 2°C and relative humidity of 55% ± 2% with a 12-hour light/dark circle. The food and water were freely accessed. All rats adapted to the environment for 1 week before experiments.

PHN model

200 μg/kg of RTX (Acros, Belgium, USA) was intraperitoneally injected to establish the PHN models. After successful modeling, the rats will show symptoms such as mechanical allodynia and thermal hypoalgesia similar to the patients with postherpetic neuralgia (PHN) (Lei et al., 2016; Pan et al., 2003). RTX induces thermal hypoalgesia by depleting primary sensory neurons that express the capsaicin receptor vanilloid receptor 1 (VR1 receptors), and the RTX-induced mechanical allodynia is probably due to the damage to both myelinated afferent nerves and their abnormal sprouting in lamina II of the spinal dorsal horn (Pan et al., 2003). RTX was dissolved in 10% Tween-80, 10% ethanol and 80% normal saline(Lei et al., 2016; Pan et al., 2003). The control group received the same volume of solvent (10% Tween 80, 10% ethanol and 80% normal saline).

Experiment design and drug administration

Twenty-eight days after RTX or RTX solvent injection, rats were randomly divided into three groups: Control group, RTX + saline group and RTX + lidocaine group. Rats of the 3 groups were anesthetized with 2-3% (vol) of isoflurane. Then the RTX + lidocaine rats were slowly (>10 min) and intravenously injected with a dose of 25 mg/kg lidocaine with a pump, while the RTX + saline rats and the control rats received the same amount of saline. Lidocaine or saline were administered continuously for 7 days.

Pain threshold tests

Before RTX injection (day 0), after RTX injection (day 2, 4, 7, 14, 21, and 28), after daily lidocaine administration, and 7 days after the last lidocaine administration, the mechanical withdrawal thresholds and thermal withdrawal latencies of the hind right paw were tested to confirm the success of modeling.

Mechanical withdrawal thresholds

Rats were individually placed on a mesh suspension plate for 30 minutes to adapt to the environment. Mechanical withdrawal thresholds were tested by an von Frey electronic pain meter (IITC, Wood Dale, IL, USA) (Cao et al., 2017). The device was used to exert a certain mechanical stimulus to the right hind paw vertically, and when the rats lifted their hind paws, the tests were completed and the mechanical withdrawal thresholds will be recorded. If the measured value exceeded 50.0 g, it was recorded as 50.0 g. Five measurements were performed on each rat with a 5-minute interval, and the average was regarded as mechanical withdrawal threshold.

Thermal withdrawal latencies

Rats were individually placed in a plexiglass enclosure on a transparent glass surface maintained at 25 °C. After adapting to the environment for 30 minutes, they were measured by a plantar radiant heat pain tester (IITC, Wood Dale, IL, USA), which consisted of a light-emitting projection lamp and an electronic timer(Khan et al.,

2002). After placing the light beam under the right hind paw vertically, the device will be activated. The time it recorded automatically before the rat lift their hind paw is exactly the thermal withdrawal latency. The cutoff time, which was used to prevent damage to the right foot of rats was set as 30 seconds. Each rat was performed 5 times with a 5-minute interval. The average of the five tests was considered thermal withdrawal latency.

Inclined plate test

Twenty-eight days after RTX injection, inclined plate test was used to assess the motor function. Rats were placed to the longitudinal axis of a plate with perpendicular body axis. The plate was gradually raised with a gradient of 5 degrees while observing whether the rat could stay on the plate for at least 5 seconds. Record the maximum angle and repeat the process three times for each rat (Yang et al., 2019).

Evaluation of anxiety and depressive-like behaviours

Four weeks after RTX injection, the open field test (OFT) and elevated plus maze (EPM) tests were used to evaluate the anxiety-like behaviours in PHN rats, while tail suspension test (TST) was used to evaluate the depressive-like behaviours. After 7 days of lidocaine administration, the anxiety- and depression-like behaviours in PHN rats were evaluated again. Smart 3.0 system (Panlab, Barcelona, Spain) was used to record the anxiety- and depressive-like behaviours. Rats acclimated to the environment for 1 hour before experiments.

Open field test (OPT)

OFT is one of the commonly used tests to evaluate anxiety-like behaviours in rats. It consists of a center zone and a peripheral zone (100 cm x 100 cm). The rats were placed in the center and allowed to explore freely for 10 minutes. The entries to the central area and time in the central area were recorded. Increased entries and extension of time in the central area were explained as exploratory behaviour, the reduction of which reflected anxiety-like behaviour(Kühne et al., 2018).

Elevated plus maze (EPM) test

EPM test is also one of the commonly used tests to assess anxiety-like behaviours in rats. It consists of two opposite open arms (50 cm × 10 cm) and two opposite closed arms (50 cm × 10 cm). The four arms are placed in a cross shape, extending from the central platform (10 cm x 10 cm) and 50 cm above the ground. The rats were placed on the central platform facing the open arms to explore the maze for 5 minutes. The entries to the open and closed arms as well as the time in the open and closed arms were recorded. Increased entries and extension of time in the open arms were interpreted as exploratory behavior, the reduction of which reflected anxiety-like behaviours(do Espírito Santo et al., 2019).

Tail suspension test (TST)

TST is one of the most commonly used tests to assess depressive-like behaviours. The rats were hung on the hook using tape. It was 6 minutes of the tail suspension time for each rat(Xie et al., 2019). Rats were allowed to acclimate to the environment in the first minute, and their immobility time was recorded in the next 5 minutes. The immobility criterion was that the rats only had breathing and beard movements. Prolonged immobility time was considered to be a manifestation of depressive-like behaviours.

Enzyme-linked immunosorbent assay (ELISA)

The tail vein blood of the rats was collected before the lidocaine administration, 3 days and 7 days after the first lidocaine administration. Blood samples were left to clot for 1 h and centrifuge at 1000 rpm, 4°C for 10 minutes. Finally, the supernatant serum was collected to detect the expression of TNF-α, IL-1β and IL-4 with ELISA kits (Elabscience, Wuhan, China) by the manufacturer's instructions. Optical density (OD) of each well at 450 nm was measured.

Immunofluorescence

One week, 2 weeks, and 4 weeks after the first lidocaine administration, the rats were

anesthetized, perfused with phosphate buffer saline (PBS) and then fixed with 4% paraformaldehyde (PFA). The spinal cords and brains were taken immediately for frozen sectioning. Then the tissue was transferred to 30% sucrose to dehydrate after the 24 h of post-fixing with 4% PFA. After sunk to the bottom, the tissue was cut into 30-μm thickness with freezing microtome (Leica, Wetzlar, Germany). Sections were washed using PBS with 0.3% Triton X-100 (5 minutes, 3 times), then blocked with 1% BSA and 0.3% Triton X-100 in the PBS for 2 hours. Then, sections were incubated with rabbit anti-IBA1 (1: 1000; Wako, Osaka, Japan) and rabbit anti-GFAP (1: 1000; Abcam, Cambridge, UK) antibody respectively, and refrigerated at 4°C overnight. After washing the primary antibody with PBS containing 0.3% Triton X-100 (5 minutes, 3 times), sections were added with secondary antibody: goat anti-rabbit IgG H & L (1: 1000; Abcam, Cambridge, UK), and incubated for 2 hours at room temperature without light. Finally, sections were attached on glass slide, mounted with DAPI Fluoromount-G (SouthernBiotech, Alabama, USA) and photographed under a fluorescence microscope (Olympus, Tokyo, Japan). Image J (NIH, Washington, USA) were used to calculate cell fluorescence proportion, cell number and cell soma sizes.

Quantitative real-time polymerase chain reaction (qRT-PCR)

After 7 days of lidocaine administration, the dorsal parts of spinal cords were used to extract the total RNA with RNAiso Plus (TaKaRa, Shiga, Japan). Then PrimeScript™ RT reagent Kit with gDNA Eraser (TaKaRa, Shiga, Japan) was used to erase genomic DNA and synthesized cDNA in the total RNA. The qRT-PCR was performed using CFX96 Real-Time PCR Detection System (Bio-Rad, California, USA) with TB Green™ Premix Ex Taq™ II (TaKaRa, Shiga, Japan) at 95℃ for 30 seconds and 40 cycles of 95℃ for 5 seconds followed by 60℃ for 30 seconds. The primer sequences were listed in Table1. The data was calculated with $2^{-\Delta\Delta Ct}$ method.

Table S1. Primer sequences, related to Figure 3

Genes		Primer sequences
TNF-α:	forward	5'-CAT GAG CAC GGA AAG CAT GA-3'
	reverse	5'-CCA CGA GCA GGA ATG AGA AGA-3'
IL-1β:	forward	5'-TTG CTT CCA AGC CCT TGA CT-3'
	reverse	5'-CTC CAC GGG CAA GAC ATA GG-3'
GAPDH:	forward	5'-ATG GCT ACA GCA ACA GGG-3'
	reverse	5'-TTA TGG GGT CTG GGA TGG-3'

Statistical analysis

Graphpad Prism 8.0 (GraphPad Software, La Jolla, CA) and SPSS 19.0 (IBM, Chicago, IL, USA) were used for statistical analysis. Data were expressed as mean ± SEM. Statistically differences between multiple groups at the same time point were analyzed by one-way ANOVA, followed by Tukey's multiple comparisons tests. $P < 0.05$ was considered statistically significant. Immunofluorescence images were analyzed statistically using Image J v.1.8 (NIH, Washington, USA).

Supplemental references

Cao, S., Deng, W., Li, Y., Qin, B., Zhang, L., Yu, S., Xie, P., Xiao, Z., and Yu, T. (2017). Chronic constriction injury of sciatic nerve changes circular RNA expression in rat spinal dorsal horn. J Pain Res *10*, 1687-1696.

do Espírito Santo, C.C., da Silva Fiorin, F., Ilha, J., Duarte, M.M.M.F., Duarte, T., and Santos, A.R.S. (2019). Spinal cord injury by clip-compression induces anxiety and depression-like behaviours in female rats: The role of the inflammatory response. Brain Behav Immun *78*, 91-104.

Kühne, S.G., Schalla, M.A., Friedrich, T., Kobelt, P., Goebel-Stengel, M., Long, M., Rivalan, M., Winter, Y., Rose, M., and Stengel, A. (2018). Nesfatin-1(30-59) injected intracerebroventricularly increases anxiety, depression-like behavior, and anhedonia in normal weight rats. Nutrients *10*, 1889.

Khan, G.M., Chen, S.R., and Pan, H.L. (2002). Role of primary afferent nerves in allodynia caused by diabetic neuropathy in rats. Neuroscience *114*, 291-299.

Lei, Y., Sun, Y., Lu, C., Ma, Z., and Gu, X. (2016). Activated glia increased the level of proinflammatory cytokines in a resiniferatoxin-induced neuropathic rain rat model. Reg Anesth Pain Med *41*, 744-749.

Liu, L.L., Li, J.M., Su, W.J., Wang, B., and Jiang, C.L. (2019). Sex differences in depressive-like behaviour may relate to imbalance of microglia activation in the hippocampus. Brain Behav Immun *81*,

188-197.
Pan, H.L., Khan, G.M., Alloway, K.D., and Chen, S.R. (2003). Resiniferatoxin induces paradoxical changes in thermal and mechanical sensitivities in rats: mechanism of action. J Neurosci *23*, 2911-2919.
Sorge, R.E., and Totsch, S.K. (2017). Sex Differences in Pain. J Neurosci Res *95*, 1271-1281.
Xie, W., Meng, X., Zhai, Y., Ye, T., Zhou, P., Nan, F., Sun, G., and Sun, X. (2019). Antidepressant-like effects of the Guanxin Danshen formula via mediation of the CaMK II-CREB-BDNF signalling pathway in chronic unpredictable mild stress-induced depressive rats. Ann Transl Med *7*, 564.
Yang, P., Chen, A., Qin, Y., Yin, J., Cai, X., Fan, Y.J., Li, L., and Huang, H.Y. (2019). Buyang huanwu decoction combined with BMSCs transplantation promotes recovery after spinal cord injury by rescuing axotomized red nucleus neurons. J Ethnopharmacol *228*, 123-131.
